# Effect of Sintering Temperature on Structural, Dielectric, and Magnetic Properties of Multiferroic YFeO_3_ Ceramics Fabricated by Spark Plasma Sintering

**DOI:** 10.3390/ma10030267

**Published:** 2017-03-07

**Authors:** Meng Wang, Ting Wang, Shenhua Song, Qing Ma, Renchen Liu

**Affiliations:** 1 Shenzhen Key Laboratory of Advanced Materials, Department of Materials Science and Engineering, Shenzhen Graduate School, Harbin Institute of Technology, Shenzhen 518055, China; wangmeng1985@hit.edu.cn (M.W.); twang-hawk@foxmail.com (T.W.); 2 Research Institute of Tsinghua University in Shenzhen, Shenzhen 518055, China; renchen.liu@gmail.com; 3 Tsinghua Innovation Center in Dongguan, Dongguan 523808, China

**Keywords:** multiferroic materials, spark plasma sintering, low-temperature solid reaction, dielectric properties, magnetic properties

## Abstract

Based on precursor powders with a size of 200–300 nm prepared by the low-temperature solid reaction method, phase-pure YFeO_3_ ceramics are fabricated using spark plasma sintering (SPS) at different temperatures. X-ray diffraction (XRD) and scanning electron microscopy (SEM) reveal that the high-purity YFeO_3_ ceramics can be prepared using SPS, while the results from X-ray photoelectron spectroscopy (XPS) show that the concentration of oxygen vacancies resulting from transformation from Fe^3+^ to Fe^2+^ is low. The relative density of the 1000 °C-sintered sample is as high as 97.7%, which is much higher than those of the samples sintered at other temperatures. The present dielectric and magnetic properties are much better than those of the samples fabricated by conventional methods. These findings indicate that the YFeO_3_ ceramics prepared by the low temperature solid reaction and SPS methods possess excellent dielectric and magnetic properties, making them suitable for potential applications involving magnetic storage.

## 1. Introduction

In recent years, a novel material coupled with two or more magnetic, electric, and elastic orders, commonly referred to as multiferroic material, has become the focus of scientific and industrial interest because of its unusual properties, with potential applications in telecommunication and memory devices. For example, second generation multiferroic materials, such as RFeO_3_ (R = Gd, Er, La, Yb, Lu, and Y), have attracted much research attention. Unlike the first generation multiferroic materials possessing high ferroelectricity and low ferromagnetism, the RFeO_3_ groups not only combine ferroelectric and anti-ferromagnetic properties but also show favorable magnetoelectric coupling effects [1,2,3,4].

The purpose of this paper is to describe a method used to fabricate YFeO_3_ ceramics by a low-temperature solid reaction method along with spark plasma sintering. YFeO_3_ is not only widely studied but also possesses antiferromagnetic nature with a high Néel temperature (*T*_N_ = 640 K) and excellent dielectric property at room temperature. Although normal ferroelectricity is not possible in YFeO_3_ due to its space group of symmetrical Pnma, it is still worthwhile to investigate its dielectric characteristics, magnetic ordering related to dielectric relaxations, and potential ferroelectricity [5,6,7,8].

However, fabrication methods restrain the preparation of high-quality YFeO_3_. Most studies adopted the conventional solid state reaction method to fabricate YFeO_3_ [9,10]. The main disadvantages of this method are: (1) the preparation process includes complex procedures, such as preheat, sintering, and heat treatment; (2) high calcination temperature and long sintering time are needed; and (3) some secondary phases are usually introduced during the process. These impurities can have a severe impact on the magnetic properties of the material. For instance, remnant magnetization and coercive field of YFeO_3_ ceramics prepared via the conventional solid-state method is as low as 0.81 emu/g and 1 kOe, respectively [10]. These levels limit the potential application of YFeO_3_ ceramics.

Another precursor fabrication candidate is the sol-gel method. Wu et al. [11], Racu et al. [12] and Shang et al. [13] successfully prepared YFeO_3_ powders by the sol-gel method and Zhang et al. [14] developed a one-step synthesis of YFeO_3_ nanocrystals via a sol-gel auto-combustion method. The main drawbacks of this method are (1) the size of the particles increases to a submicron scale and (2) when the powders are used to prepare ceramics, many pores are introduced, resulting in a material with relatively low density [9,10,15,16]. So a simple and high efficiency method should be developed for YFeO_3_ powder preparation.

In view of this situation, a method for precursor fabrication, namely the low-temperature solid reaction method, has the advantage of easy phase control, pure phase production, low cost, and ultrafine particle size. It only needs grinding in an agate mortar to fabricate precursors, so the procedure is greatly simplified. This method was successfully adopted to obtain YMnO_3_ and ZnFe_2_O_4_ precursors [17]. Therefore, this method may be used to synthesize fine YFeO_3_ precursor powders.

Recently, spark plasma sintering (SPS) has been aggressively adopted as an advanced method for powder metallurgy fabrication. One notable feature of SPS is that it allows the use of a low sintering temperature (200–300 K lower than that of the conventional method) and a short sintering time (typically only 5 min), so that high-density structures can be obtained [18,19,20,21]. Ma et al. have developed dense Y(Mn,Fe)O_3_ and YMnO_3_ nano-size ceramics [22,23]. Song et al. and Wang et al. obtained BiFeO_3_ nano-ceramics using precursors prepared by ball milling and sol-gel methods, respectively [24,25]. However, it should be pointed out that even using the SPS method, the impure phase still exists and the grain size still needs to be precisely controlled.

Two important aspects of the study are emphasized. They include an SPS fabrication procedure with a low-temperature solid reaction precursor preparation method along with annealing in air to produce the YFeO_3_ ceramic and the sintering temperatures for SPS processes. The microstructures of YFeO_3_ precursor and ceramics were studied extensively. The dielectric characteristics of YFeO_3_ ceramics were evaluated over broad temperature and frequency ranges. The relationship between the magnetic properties and microstructures of the fabricated YFeO_3_ samples was also studied.

## 2. Experimental Procedures

In this study, YFeO_3_ powders were synthesized via the low-temperature solid reaction method. The raw reagents include Fe(NO_3_)_3_∙9H_2_O, Y(NO_3_)_3_∙6H_2_O, and citric acid. Initially, Fe(NO_3_)_3_∙9H_2_O, Y(NO_3_)_3_∙6H_2_O and citric acid under a mole ratio of 1:1:2 were weighed and ground in an agate mortar for half an hour, respectively. Then the respective powders were mixed and ground again in an agate mortar for half an hour. A light brown viscous substance was formed during the grinding process, implying that the complex was formed. The viscous substance was heated at 120 °C for 2 h to remove free water and then a powdery composite was harvested, serving as the precursor material. The powders were ground and subsequently calcined for 1 h in air at 800 °C to provide what shall be referred to as the pure phase YFeO_3_ powders, serving as raw precursors for the subsequent SPS process. In the SPS process, the YFeO_3_ powders were placed in a graphite die and heated at a rate of 100 °C/min from room temperature to 900, 950, 1000, and 1050 °C, respectively, followed by sintering for 5 min under an atmospheric pressure of 10^−2^ Pa. During the entire SPS process, a uniaxial pressure of 50 MPa was constantly maintained to the sample [26]. After completing the sintering process, pellet-shaped samples were formed. The samples were then polished to a size of 2 mm thick and 10 mm diameter, followed by annealing at 800 °C for 2 h in air in order to recover the oxygen stoichiometric ratio, release strain, and remove carbon contamination.

The crystal structures of the pellet samples were analyzed using X-ray Diffraction (XRD) (D/max-RB, Rigaku, Tokyo, Japan). The fracture surfaces of the samples were observed using scanning electron microscopy (S-4700, Hitachi, Tokyo, Japan). The densities of the pellets were measured via traditional Archimedes method with distilled water. X-ray photoelectron spectroscopy (XPS) measurements were carried out on an ESCALAB 250Xi system (ESCALAB 250Xi, Perkin Elmer, Waltham, MA, USA) with Mg Kα radiation. The XPS curves were fitted with XPS-Peakfit software through Gauss-Lorentz line. All the samples were then polished to 1 mm thick, sprayed with silver paint, and cured for 1 h at 120 °C. This procedure was required in order to prepare the test samples for dielectric measurements. Magnetic properties of the samples were obtained using a physical property measurement system (DynaCool-9T, Quantum Design, Leatherhead, Surrey, UK) at room temperature.

## 3. Results and Discussion

**Figure 1:**
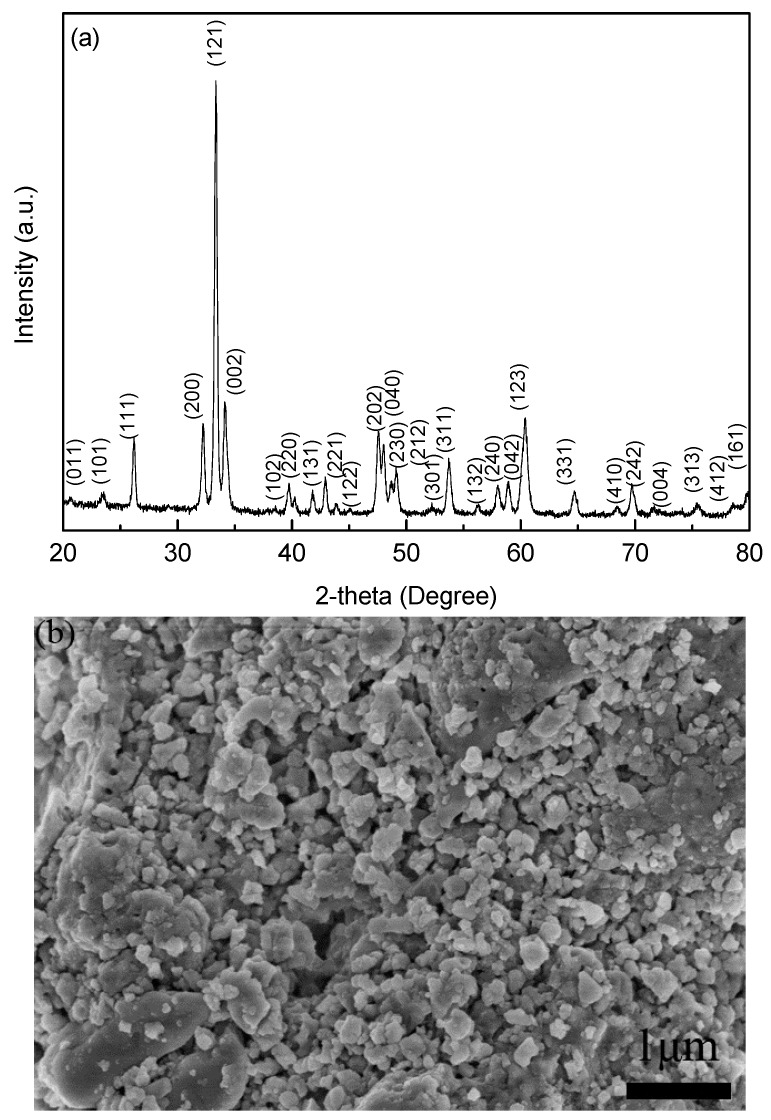
(**a**) XRD pattern and (**b**) SEM micrograph for YFeO_3_ powders.

Figure 1a reveals the XRD pattern of the precursor powders prepared via the low-temperature solid reaction method. Clearly, the diffraction peaks are well-fitted to the YFeO_3_ phase possessing an orthorhombic perovskite structure with space group of Pnma. No visible impure phases (such as Y_2_O_3_, Fe_2_O_3_, and Y_3_Fe_5_O_12_) can be detected in the powders within the limits of the XRD machine capability. Consequently, high-purity powders are successfully obtained. Figure 1b shows the SEM micrograph of the YFeO_3_ precursor powders. The particles appear to be homogeneous in the size range of ~ 200–300 nm.

**Figure 2:**
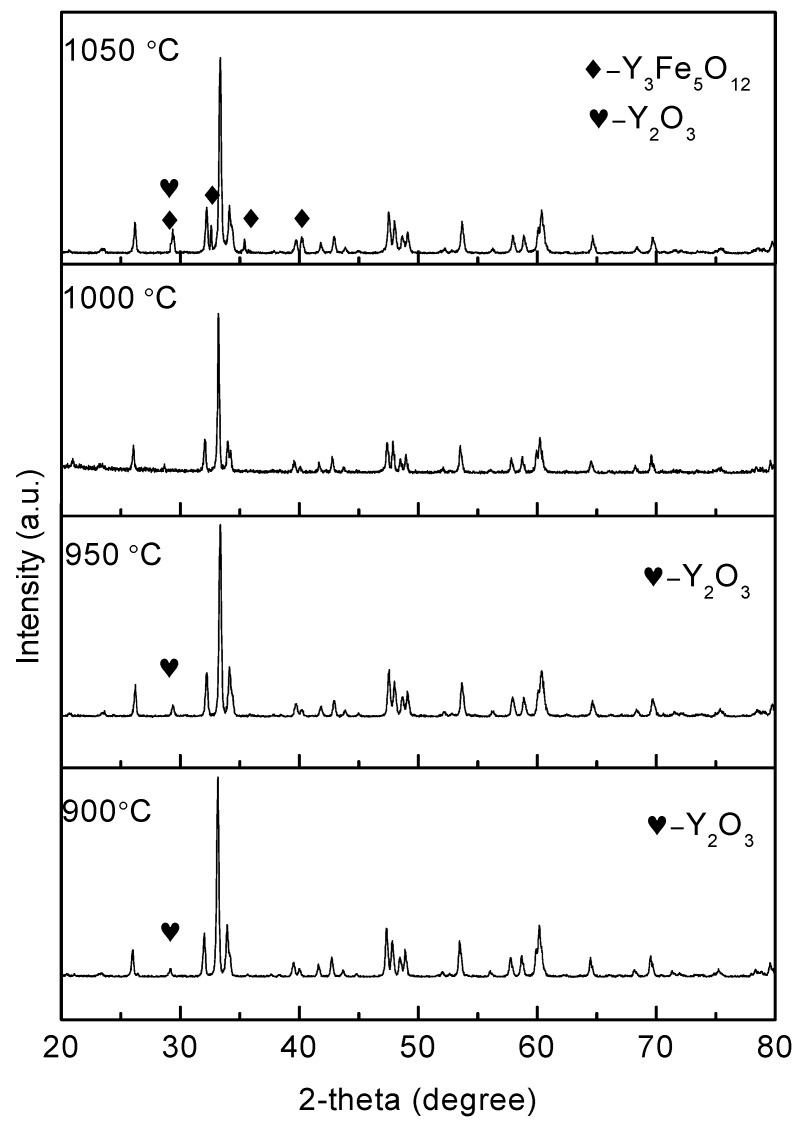
XRD patterns for YFeO_3_ samples sintered at different temperatures (900, 950, 1000, and 1050 °C).

XRD patterns of the SPS-prepared samples sintered at different temperatures are shown in Figure 2. The unique high-purity phase pattern is presented in the 1000 °C-sintered sample. As for the 900 °C and 950 °C-sintered samples, the secondary phase Y_2_O_3_ (heart shaped symbol) can be detected. The transformation of Y_2_O_3_ + Fe_2_O_3_ ↔ 2YFeO_3_ is common in the range of 900–1000 °C. The pattern for the 1050 °C-sintered sample suggests the presence of Y_3_Fe_5_O_12_. The transformation from the orthorhombic perovskite YFeO_3_ to Y_3_Fe_5_O_12_ and Y_2_O_3_ (5YFeO_3_ ↔ Y_3_Fe_5_O_12_ + Y_2_O_3_) is normal around 1100 °C, and it is probably because an extra energy is supplied by SPS. Accordingly, this transformation can occur in the 1050 °C-sintered sample. This behavior is in accordance with the finding reported in [27]. In summary, the unique YFeO_3_ phase without impurities can only be acquired for the 1000 °C-sintered sample, which is 300–400 °C lower compared to conventional methods.

**Figure 3:**
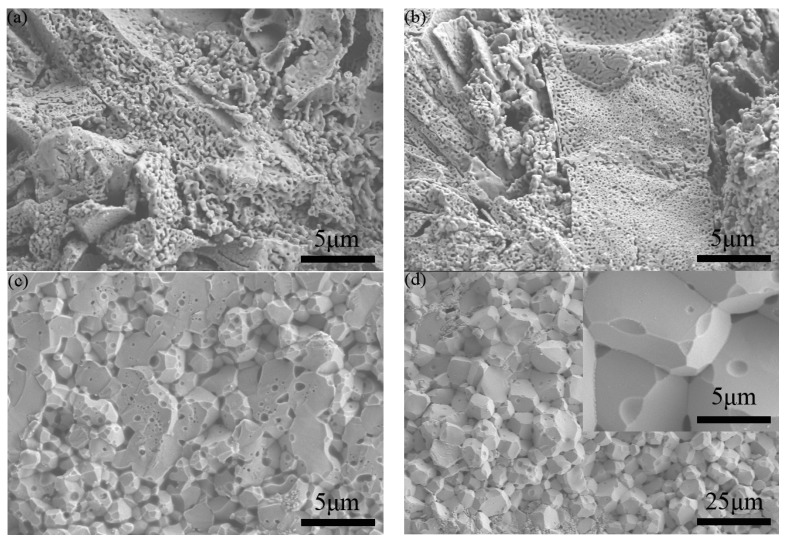
SEM micrographs of fracture surfaces of the YFeO_3_ ceramics sintered at different temperatures: (**a**) 900 °C; (**b**) 950 °C; (**c**) 1000 °C; (**d**) 1050 °C.

SEM micrographs of the fracture surfaces of the samples sintered at four different temperatures are shown in Figure 3. Normally, the fracture surface, rather than thermally etched polished cross-sections of samples, is a good way to present the real morphology and defects in ceramics. For the cases of sintering at 900 °C and 950 °C, the samples show large cracks and pores, proving that these samples were not successfully sintered. Meanwhile, the 1000 °C- and 1050 °C-sintered samples exhibit a high-density YFeO_3_ structure with smooth facets. After the SPS sintering and annealing process, the grain size of the sintered ceramics increases to ~ 1–2 μm for the 1000 °C-sintered sample and ~ 5–8 μm for the 1050 °C-sintered sample. The increase in grain size is due to the higher sintering temperature (1000–1050 °C) during the SPS process compared with the temperatures used in the calcining process of YFeO_3_ powders (800 °C). As for the 1050 °C-sintered sample, the lower densities of impurities Y_3_Fe_5_O_12_ and Y_2_O_3_ can lead to a volume expansion at the elevated sintering temperature. These effects can result in a surface with more pores and defects [28].

**Figure 4:**
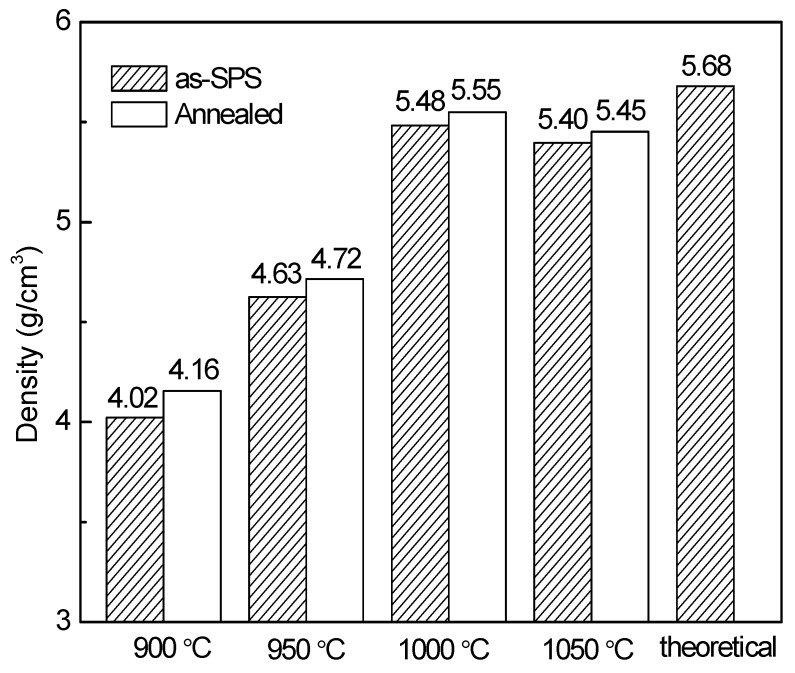
The densities of the samples after SPS and annealing.

The densities of the YFeO_3_ pellet samples are shown in Figure 4. Before annealing, the density of the 1000 °C-sintered sample reaches its highest value of 5.484 g/cm^3^. This value is equivalent to a relative density of 96.5% (theoretical density = 5.68 g/cm^3^). For the 900 °C and 950 °C-sintered ceramics, the densities of 4.023 g/cm^3^ and 4.625 g/cm^3^ are obtained, respectively. These are only 70.8% and 81.4% of the theoretical density, suggesting that the sintering process is not fully completed. For the 1050 °C-sintered sample, the density is slightly lower than that of the 1000 °C-sintered sample because the secondary phase Y_3_Fe_5_O_12_ and Y_2_O_3_ are introduced and more defects exists [9,29]. After annealing, all the samples exhibit slightly higher densities as compared to those before annealing, probably because the deficiency of oxygen is compensated and the carbon contamination is eliminated during the annealing treatment. This outcome suggests that, under a simultaneous use of constant uniaxial pressure and high-density heat of plasma, YFeO_3_ ceramics with high quality and density can be successfully prepared. Therefore, it is affirmed that the highly pure and dense YFeO_3_ ceramics with fine-grained structures can be fabricated using precursor powders by means of SPS at 1000 °C plus annealing at 800 °C for 2 h in air.

**Figure 5:**
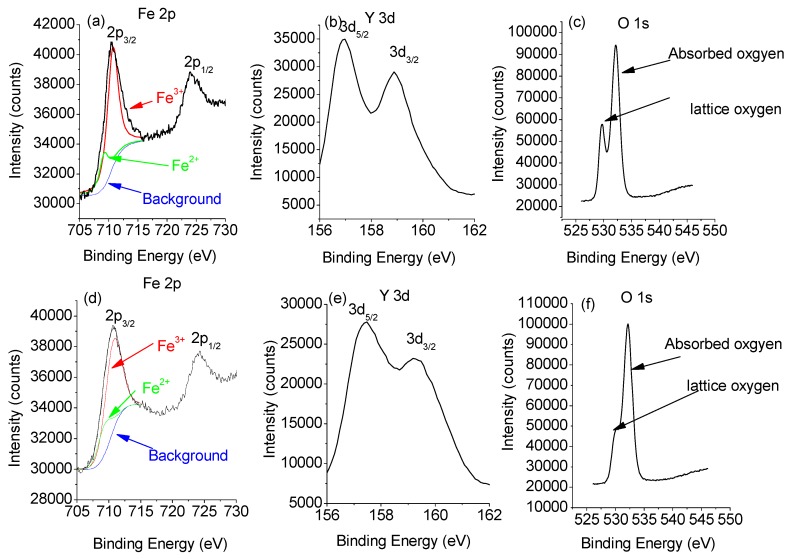
XPS spectra of the YFeO_3_ ceramics: (**a**) Y 3d; (**b**) Fe 2p; and (**c**) O 1s for 1000 °C-sintered sample; (**d**) Y 3d; (**e**) Fe 2p; and (**f**) O 1s for 1050 °C-sintered sample.

Because the 900 °C- and 950 °C-sintered samples are not fully sintered, the 1000 °C- and 1050 °C-sintered samples will be only analyzed further. Since the ionic valence has an important effect on the dielectric and magnetic properties of materials, XPS spectra are determined on the YFeO_3_ ceramics. Figure 5 depicts the Y 3d, Fe 2p, and O 1s XPS patterns of the 1000 °C- and 1050 °C-sintered YFeO_3_ samples. Binding energy (BE) values are 157.2 eV and 159.0 eV for Y 3d_3/2_ and Y 3d_5/2_, respectively, and ΔY 3d = 1.8 eV is in good agreement with the published results [30]. The Fe 2p_3/2_ peak, using Gauss-Lorentz fitting, is divided into two parts representing the coexistence of Fe^3+^ and a low percentage of Fe^2+^. The binding energies for Fe^2+^ 2p_3/2_ and Fe^3+^ 2p_3/2_ are 709.20 and 710.70 eV, respectively. These values not only agree well with the values presented in the published reports but validate the coexistence of Fe^2+^ and Fe^3+^ [12]. Evaluation of the peak spectra regions shows that the peak for Fe^2+^ ions is less than 10% of the entire Fe component. The peak for Fe^2+^ ions in the 1000 °C-sintered sample is 6.2% of the entire peak, while that in the 1050 °C-sintered sample reaches 8.9%. The reason for the emerging Fe^2+^ ions in the YFeO_3_ sample is attributed to the presence of oxygen vacancies, which is unavoidable in RFeO_3_ ceramic materials. However, the concentration of these ions is reasonably low. For the O 1s XPS pattern, two peaks are shown, namely one for lattice oxygen (O^2−^) at 528 eV and the other for surface absorbed oxygen (O^−^) at 532 eV [31]. The pattern shows that the height of the lattice oxygen (O^2−^) peak is lower than that of the surface absorbed oxygen (O^−^) peak. Also, both peak height and area of the lattice oxygen peak of the 1050 °C-sintered sample are much lower than those of the 1000 °C-sintered sample. This is likely due to the formation of oxygen vacancies. The formation of an oxygen vacancy can lead to the loss of an electron in the oxygen ion, resulting in a transfer from O^2−^ to O^−^ [32]. Therefore, it can be concluded that the concentration of oxygen vacancies in the 1000 °C-sintered sample is lower than that in the 1050 °C-sintered sample. This phenomenon can affect the dielectric and magnetic properties of materials. Also, the above analysis shows that it is possible to fabricate high-purity YFeO_3_ at 1000 °C.

**Figure 6:**
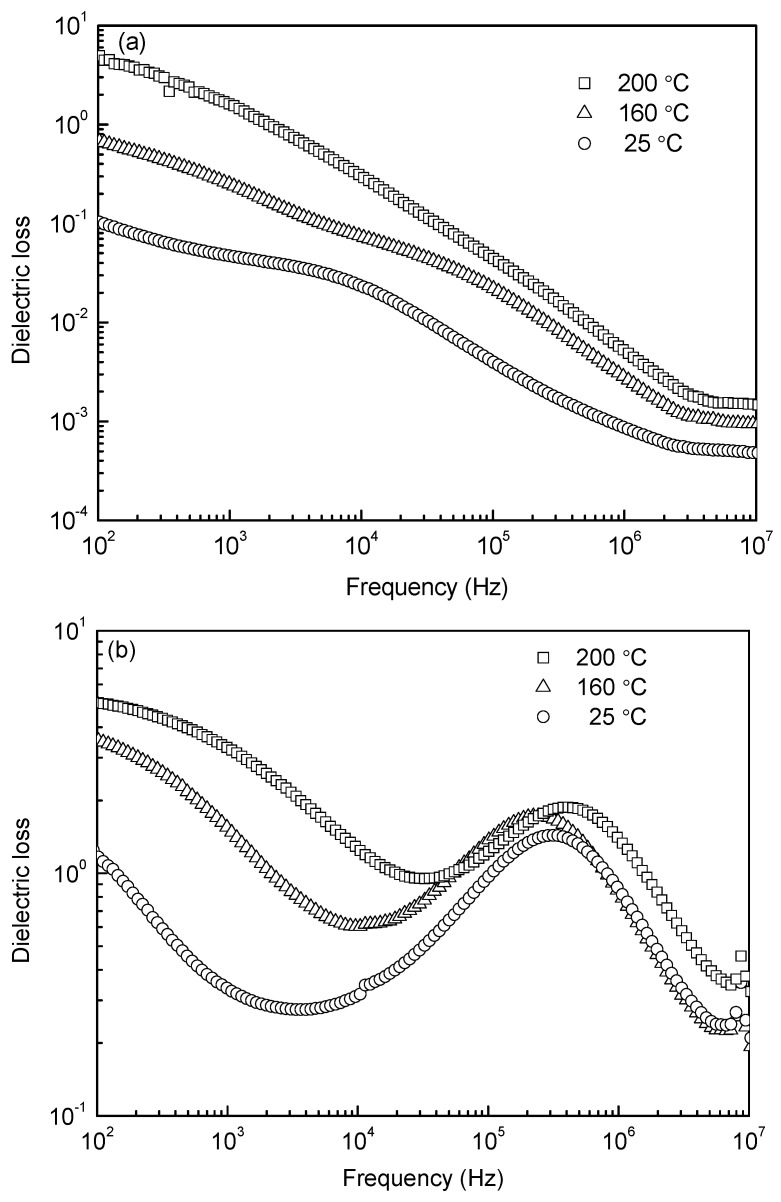
Frequency dependences of dielectric loss at different temperatures for the samples sintered at (**a**) 1000 °C and (**b**) 1050 °C.

**Figure 7:**
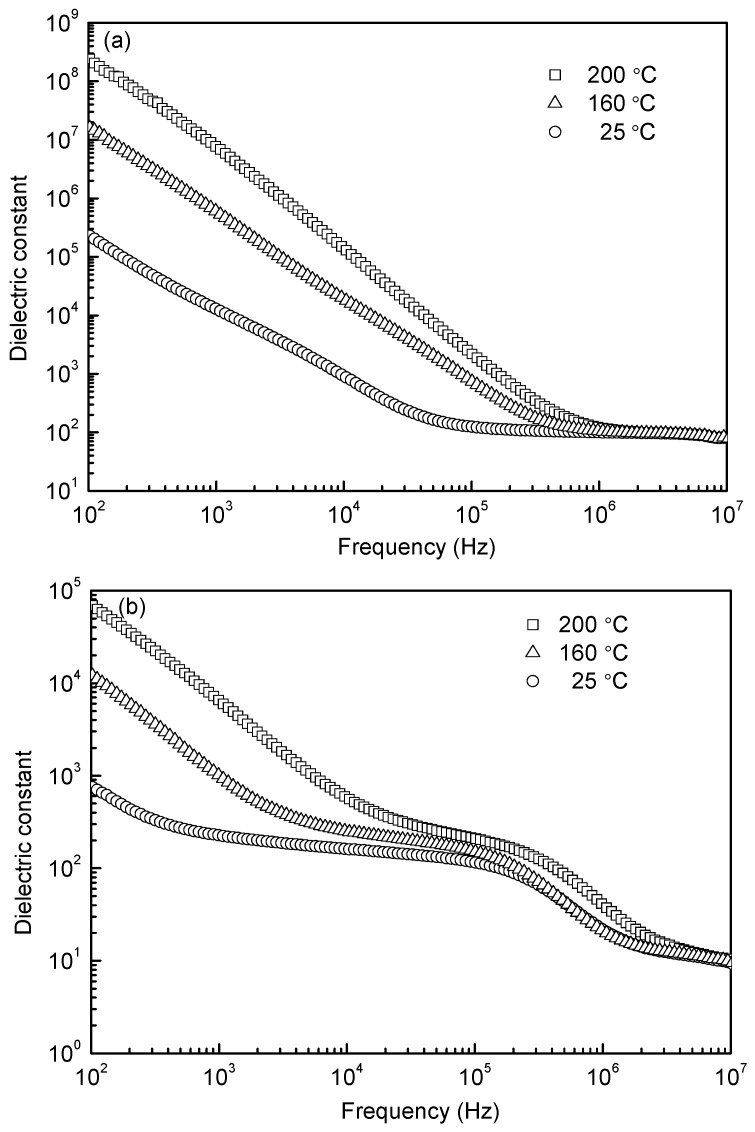
Frequency dependences of dielectric constant at different temperatures for the samples sintered at (**a**) 1000 °C and (**b**) 1050 °C

Figure 6 shows the frequency dependences of dielectric loss at different temperatures for the samples sintered at 1000 °C and 1050 °C. Both diagrams (Figure 6a,b) show large values of dielectric loss in low frequency, which is due to the different conductivity between grain and grain boundary. With increasing frequency, the dielectric loss exhibits a gradual decrease for the 1000 °C-sintered sample, while for the 1050 °C-sintered sample, a peak is present from 10^5^ to 10^6^ Hz, showing the dielectric relaxation. As is commonly known, each polarization mechanism is characterized by a relaxation frequency corresponding to the maximal phase shift between the polarization and the applied electric field; thus a peak of dielectric loss occurs. The peak in Figure 6b is ascribed to space charge polarization. Usually, the space charge polarization is related to oxygen vacancies, as evidenced by XPS analysis in Figure 5. Because few oxygen vacancies emerged in the 1000 °C-sintered sample, no peak of dielectric loss can be seen. The dielectric constants as a function of frequency at room (25 °C) and elevated temperatures (160 °C and 200 °C) for the 1000 °C- and 1050 °C-sintered samples are shown in Figure 7. Both samples have a high value in the lower frequency range and drop drastically with increasing frequency above a turning point. The high values are associated to the high conductivity, the Maxwell-Wagner relaxation, which is often observed in heterogeneous systems with different conductivities. As the frequency increases, these factors are significantly damped out so that the space charges fail to comply with changes in the frequency of electrical field and result in a reduction in dielectric constant [33,34]. The dielectric constant shows a sharp decrease with increasing frequency in the low frequency range and a plateau in the high frequency region. Note that the plateau moves to higher frequencies with increasing temperature, which is an effect due to the Debye type thermally activated mechanism [22]. As shown in Figure 7, there is one plateau for the 1000 °C-sintered sample, but there are two for the 1050 °C-sintered sample, the first one of which is just corresponding to the relaxation peak shown in Figure 6b. This could be because the 1050 °C-sintered sample possesses more defects, such as pores and oxygen vacancies, as the space charges are related to the defects, thereby resulting in a different polarization phenomenon. In addition, the dielectric constant is apparently higher for the 1000 °C-sintered sample, which is due to the lower oxygen vacancies, smaller grain size, and higher density of the sample (see Figure 3, Figure 4 and Figure 5, respectively).

**Figure 8:**
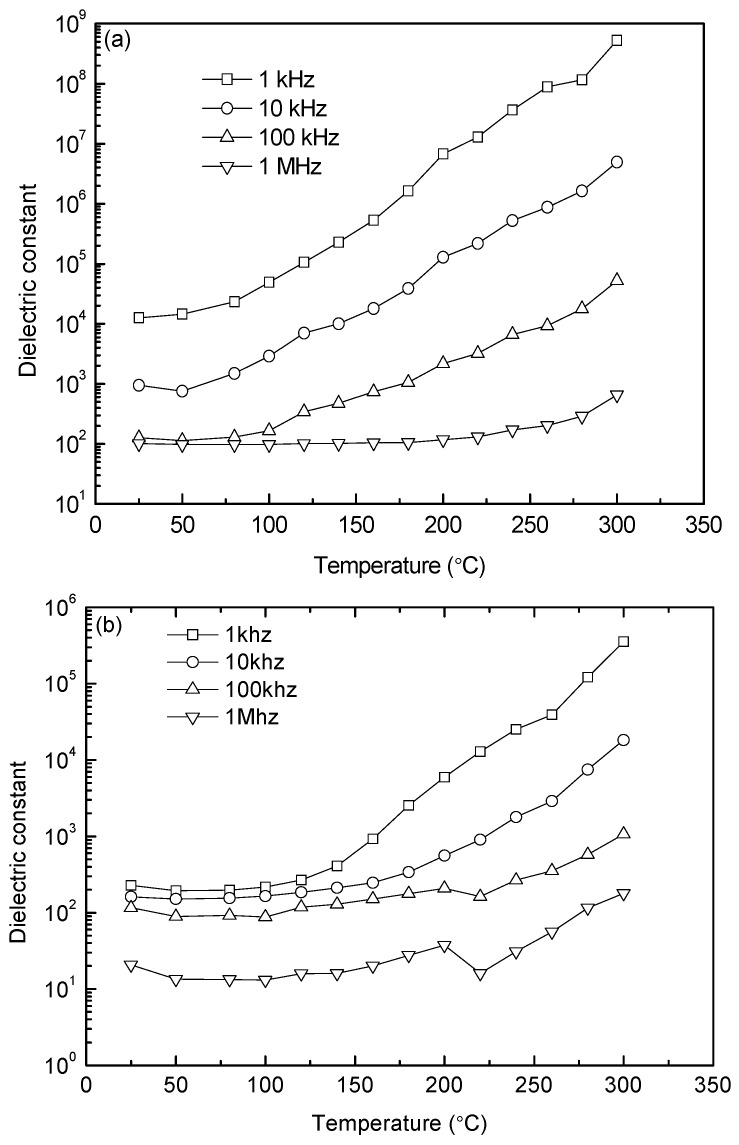
Temperature dependences of dielectric constant under frequencies of 1, 10, 100, and 1000 kHz for the samples sintered at (**a**) 1000 °C and (**b**) 1050 °C.

Figure 8 shows the temperature dependences of dielectric constant at the frequencies of 1 kHz, 10, 100, and 1 MHz for the 1000 °C- and 1050 °C-sintered samples. At a given temperature, the value of dielectric constant decreases with increasing frequency. In addition, the dielectric constant remains almost constant until 100 °C, and then starts to increase with further increasing temperature. An obvious dielectric relaxation effect can be observed in the range of 100–300 °C. Also note that this effect is delayed to higher temperatures as the frequency increases. The dielectric constant increases with increasing temperature and it is attributed to the conductivity enhancement. The overall dielectric constant values of the 1000 °C-sintered sample are much higher than those of the 1050 °C-sintered sample at 1 MHz (as higher frequencies can reflect the intrinsic dielectric constant of YFeO_3_). For example, the dielectric constant of the 1000 °C-sintered sample at 1 MHz and 25 °C is 100, while that of the 1050 °C-sintered sample is about 20. The former is nearly five times higher than the latter, which can be ascribed to high resistance, lower oxygen vacancies, smaller grain size, and phase purities. An anomaly in dielectric constant for the 1050 °C-sintered sample, such as a sudden decrease, is also caused by the movement and concentration of point defects. This phenomenon does not exist in the 1000 °C-sintered sample probably because of the higher density and fewer pores of the sample, in addition to the constraint of grain boundaries on the mobility of these defects [35].

**Figure 9:**
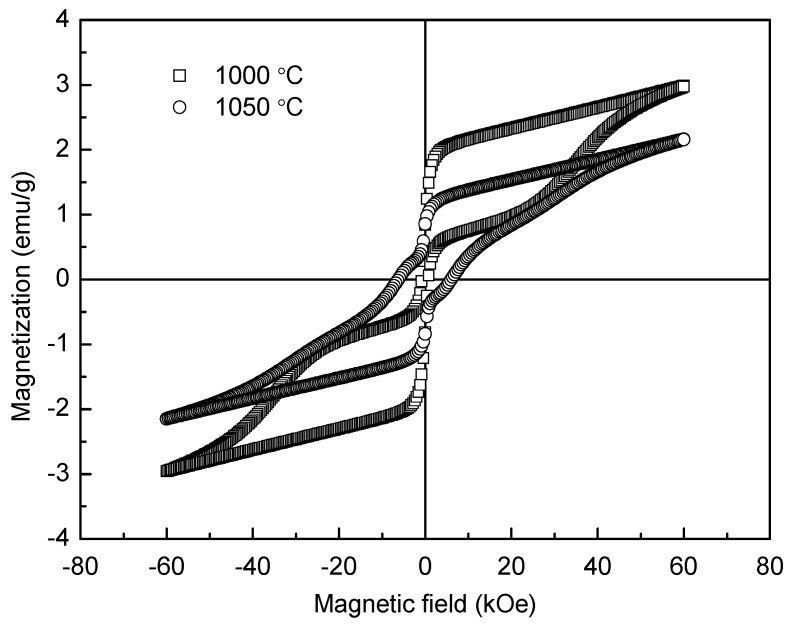
Magnetic hysteresis loops of the YFeO_3_ ceramic samples sintered at 1000 °C and 1050 °C.

Room-temperature magnetic hysteresis loops of the YFeO_3_ ceramic samples sintered at 1000 °C and 1050 °C are shown in Figure 9. It is well known that the ionic structure determines the anti-ferromagnetic property of YFeO_3_. Here a Fe^3+^ ion is surrounded by six O^2−^ ions, forming a FeO_6_ octahedra, and each O^2−^ is the shared apex of two adjacent octahedras, serving as a super exchange interaction channel [36]. According to the Dzyaloshinski-Moriyaanti symmetric exchange mechanism, each Fe^3+^ magnetic moment is located not totally parallel to the moments of all six nearest Fe^3+^ neighboring moments, thus forming a small angle [37,38]. This leads to the generation of weak ferromagnetic characteristics in antiferromagnetic YFeO_3_ ceramics and increases with the refinement of grain size. Because the grains of the 1000 °C-sintered sample are smaller than those of the 1050 °C-sintered one, the magnetic properties of the 1000 °C-sintered sample, such as *M*_m_ (maximum magnetization) and *M*_r_ (remnant magnetization), are higher. Both the 1000 °C and 1050 °C-sintered samples exhibit anti-ferromagnetic characteristics. The *M*_m_, *M*_r_, and *H*_c_ (coercive field) are about 3.00 emu/g, 0.91 emu/g, and 790 Oe, respectively, for the 1000 °C-sintered sample, while these are about 2.16 emu/g, 0.86 emu/g, and 6150 Oe for the 1050 °C-sintered sample. The *H*_c_ value of the 1000 °C-sintered sample is smaller while its *M*_m_ and *M*_r_ values are larger compared to the 1050 °C-sintered sample. In order to make a comparison, some relevant magnetic parameters of YFeO_3_ are presented in Table 1 [9,10,11,14,38,39]. Obviously, the *M*_m_ value of the 1000 °C-sintered sample is much larger than that in the reported data. Moreover, the coercive field (*H*_c_) is the same order of magnitude as that of the YFeO_3_ sample prepared by Ma et al. [10]. Compared with the results of Ma et al. and other reports [10,40], the present values of *M*_r_ and *M*_m_ are much higher than those of the samples fabricated by a conventional sintering method because of the smaller grain size and less impurity.

## 4. Conclusions

Briefly summarizing the work performed and the results obtained, the high-density impurity-free YFeO_3_ ceramics with grain sizes in the 1–2 μm range were prepared by combining a low-temperature solid reaction and spark plasma sintering at 1000 °C, along with annealing in air at 800 °C for 2 h. However, the samples sintered at 900, 950, and 1050 °C were found to possess a lower density and small amounts of impurity. The relative density of the 1000 °C-sintered sample was as high as 97% while that of the 1050 °C-sintered sample was just about 95%. The XPS showed that the Fe^3+^ ions took up the majority of Fe ions with a low concentration of Fe^2+^ ions while the 1050°C-sintered sample experienced an increase in the concentration of oxygen vacancies during the fabrication procedure. Finally, the dielectric behavior was impacted by the sintering temperature. The dielectric constant of the 1000 °C-sintered sample was found to be much higher than that of the 1050 °C-sintered sample. The higher *M*_m_ and *M*_r_ values of the 1000 °C-sintered sample were attributed to the refined grain size and high purity composition. In summary, one can conclude that the YFeO_3_ ceramics prepared by combining low-temperature solid reaction and spark plasma sintering as described in this report have excellent dielectric and magnetic properties.

## Figures and Tables

**Table 1:** Comparison of the relevant magnetic parameters for YFeO_3_ ceramics.

No.	*M*_m_ (emu/g)	*H*_c_ (kOe)	Reference
1	1.9@7.0 T	24.6	[9]
2	0.46@0.65 T	0.25	[11]
3	0.15@0.25 T	0.97	[38]
4	0.45@0.35 T	0.25	[14]
5	2.1@5 T	1.0	[10]
6	3.0@6 T	0.91	Present work
